# PET imaging to non-invasively study immune activation leading to antitumor responses with a 4-1BB agonistic antibody

**DOI:** 10.1186/2051-1426-1-14

**Published:** 2013-08-27

**Authors:** Helena Escuin-Ordinas, Mark W Elliott, Mohammad Atefi, Michelle Lee, Charles Ng, Liu Wei, Begoña Comin-Anduix, Encarnacion Montecino-Rodriguez, Earl Avramis, Caius Radu, Leslie L Sharp, Antoni Ribas

**Affiliations:** 1 Department of Medicine (Division of Hematology-Oncology) at David Geffen School of Medicine, University of California Los Angeles (UCLA), Los Angeles, USA; 2 Pfizer Worldwide Research and Development, Oncology Research Unit, San Diego, CA, USA; 3 Ahmanson Translational Imaging Division, Department of Molecular and Medical Pharmacology, UCLA, Los Angeles, USA; 4 Department of Surgery (Division of Surgical-Oncology), UCLA, Los Angeles, USA; 5 Jonsson Comprehensive Cancer Center (JCCC), Los Angeles, USA; 6 Department of Pathology and Laboratory Medicine, UCLA, Los Angeles, USA; 7 Current address: Genomics Institute of the Novartis Research Foundation, 10675 John Jay Hopkins Dr., San Diego, CA 92121, USA; 8 Department of Medicine, Division of Hematology-Oncology, 11-934 Factor Building, Jonsson Comprehensive Cancer Center at UCLA, 10833 Le Conte Avenue, Los Angeles, CA 90095-1782, USA

**Keywords:** 4-1BB, CD137, Immune activating antibodies, PET imaging, Colon cancer

## Abstract

**Background:**

Molecular imaging with positron emission tomography (PET) may allow the non-invasive study of the pharmacodynamic effects of agonistic monoclonal antibodies (mAb) to 4-1BB (CD137). 4-1BB is a member of the tumor necrosis factor family expressed on activated T cells and other immune cells, and activating 4-1BB antibodies are being tested for the treatment of patients with advanced cancers.

**Methods:**

We studied the antitumor activity of 4-1BB mAb therapy using [^18^ F]-labeled fluoro-2-deoxy-2-D-glucose ([^18^ F]FDG) microPET scanning in a mouse model of colon cancer. Results of microPET imaging were correlated with morphological changes in tumors, draining lymph nodes as well as cell subset uptake of the metabolic PET tracer *in vitro*.

**Results:**

The administration of 4-1BB mAb to Balb/c mice induced reproducible CT26 tumor regressions and improved survival; complete tumor shrinkage was achieved in the majority of mice. There was markedly increased [^18^ F]FDG signal at the tumor site and draining lymph nodes. In a metabolic probe *in vitro* uptake assay, there was an 8-fold increase in uptake of [^3^H]DDG in leukocytes extracted from tumors and draining lymph nodes of mice treated with 4-1BB mAb compared to untreated mice, supporting the *in vivo* PET data.

**Conclusion:**

Increased uptake of [^18^ F]FDG by PET scans visualizes 4-1BB agonistic antibody-induced antitumor immune responses and can be used as a pharmacodynamic readout to guide the development of this class of antibodies in the clinic.

## Background

Agonistic antibodies to 4-1BB (CD137) are in clinical testing as immunotherapy for cancer [1-3]. 4-1BB is a surface glycoprotein that belongs to the tumor necrosis factor receptor superfamily (TNFRSF). It was first identified on activated T lymphocytes [4], and has subsequently been described on other activated immune cells, including dendritic cells (DC), natural killer cells (NK), natural killer T cells (NKT), monocytes, neutrophils, eosinophils, mast cells and regulatory T cells [4]. Its ligand, CD137L or 4-1BBL, is mainly expressed on hematopoietic stem cells, antigen-presenting cells (APCs) and myeloid progenitor cells [5]. Agonistic 4-1BB mAbs induce regression of established solid tumors in several animal models by promoting T-cell survival, proliferation, cytokine production, and enhance T-cell cytolytic activity [2,6]. 4-1BB mAbs also induce tumor T-cell infiltration by stimulating the expression of cellular adhesion molecules on tumor endothelial cells [7]. While initial clinical trials are in progress [2], new promising pre-clinical studies combining this potent antibody with other therapies are also emerging [5,8] together with next generation versions of 4-1BB mAbs [9].

Positron emission tomography (PET) is a broadly available imaging method used for the *in vivo* visualization of cellular processes at a molecular level. The widely exploited [^18^ F]-labeled fluoro-2-deoxy-2-D-glucose ([^18^ F]FDG) tracer is used as a highly sensitive imaging tool that detects cells based on their increased glucose metabolism resulting from the intracellular trapping of the tracer. The most common clinical use of [^18^ F]FDG PET scanning is for the diagnosis and treatment monitoring in patients with cancer thanks to the Warburg effect [10,11]. In addition to cancer cells, activated lymphocytes and macrophages have a markedly increased glucose metabolism resulting in increased [^18^ F]FDG uptake and intracellular accumulation. Therefore, [^18^ F]FDG PET imaging may be useful in studying tumor immunotherapy [12]. In the present study we used [^18^ F]FDG microPET imaging to visualize 4-1BB agonistic antibody-induced immune cell responses within the tumor site in a colon carcinoma mouse model. Our results support the use of this PET-based immune detection as a pharmacodynamic readout to guide the development of this class of antibodies in the clinic by monitoring T cell activation after 4-1BB mAb therapy.

## Results

### Antitumor effects of 4-1BB agonistic antibodies in a murine model of colon carcinoma

**Figure 1:**
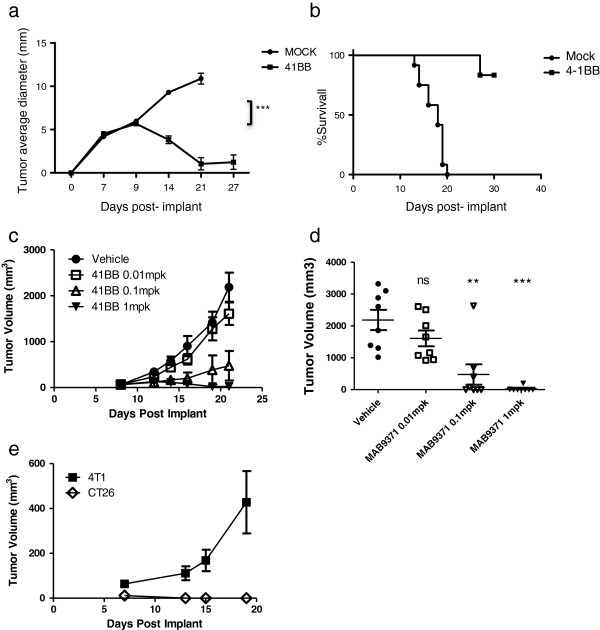
**Antitumor activity of 4-1BB mAb therapy against CT26 colon carcinoma. a)** The administration of a double dose of 4-1BB mAb (1 mg/kg) induced reproducible CT26 tumor regressions (n = 12 mice per group; 3.8 ± 0.4 4-1BB mAb group vs 9.2 ± 0.3 control, on day 14 post-tumor implant; p < 0.0001 by two-way Anova). **b)** Kaplan-Meier plot of improved survival in mice (n = 12 mice per group; p = 0.0001 for tumor diameters by two-way Anova and p = 0.0001 for survival by log rank test). **c and d)** Different 4-1BB mAb doses were also tested showing a significant tumor growth inhibition by day 21 in animals receiving 0.1 or 1 mg/kg 4-1BB mAb (ns = not significant * P < 0.05, ** P < 0.01, *** P < 0.001 by t-test). **e)** Re-challenge of surviving tumor-free mice 55 days after complete tumor shrinkage showed a total CT26 tumor rejection by the animals. 4 T1 cells were used as positive control.

We set up a model of reproducible immune-mediated tumor regressions to then allow PET-based imaging to study pharmacodynamic effects. In replicate experiments we tested the antitumor activity of a commercially available rat-anti-mouse 4-1BB mAb against the implantable CT26 colon carcinoma in fully immunocompetent mice. The administration of two 1 mg/kg doses of 4-1BB mAb induced reproducible CT26 tumor regressions (p < 0.0001 by two-way Anova) and improved survival in mice (p = 0.0001 by log rank test). All mice responded to the treatment and complete tumor shrinkage was achieved in over 80% of mice 14 days after 4-1BB mAb administration (Figure 1a,b). In order to better understand the relationship between dose and efficacy, dose range finding studies were performed. Doses of 1, 0.1 and 0.01 mg/kg of 4-1BB mAb were tested. Significant tumor growth inhibition (TGI) was observed in animals receiving a single dose of 0.1 mg/kg or 1 mg/kg (Figure 1c,d). At day 55 post-tumor implantation, seven surviving tumor-free animals from the 1 mg/kg treated group, which had complete tumor shrinkage by day 21, were re-challenged with CT26 cells and the tumor implants monitored for 19 days. These mice had been tumor- free for 36 days prior to re-challenge. Control 4 T1 cells were also implanted subcutaneously on the right flank of the mice. The CT26 implant was completely rejected, while the 4 T1 cell implant grew progressively (Figure 1e), demonstrating the induction of a tumor-specific therapeutic immune response.

### 4-1BB agonistic treatment increases tumor infiltrating leukocytes

**Figure 2:**
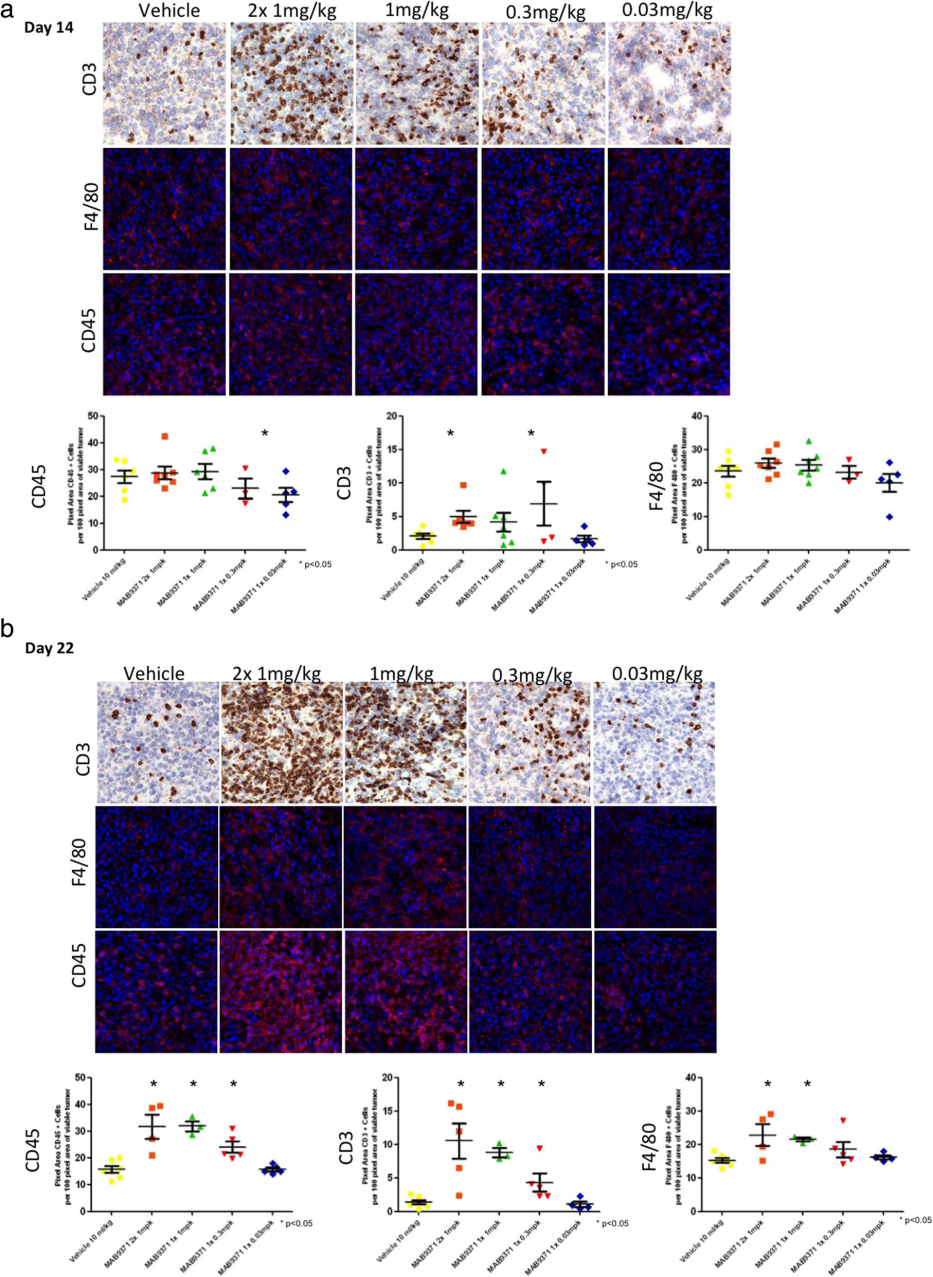
**Pathological analysis of immune infiltration after 4-1BB mAb therapy.** Tumor CD3+, F4/80+ and CD45+ infiltrating cells were identified by immunohistochemical and immunoflurecence analysis on day 14 **(a)** and day 22 **(b)** after tumor injection. Data is also represented by pixel area of positively stained cells per 100 pixel area of viable tumor. Error bars represent mean ± SEM, *p < 0.05.

In order to understand the anti-tumor immune response generated following treatment with 4-1BB antibodies, tumors from treated and control mice were extracted and processed for immunohistochemical and immunofluorescence analysis at 14 and 22 days after the start of the study. Tumor infiltrating CD45+ leukocytes, CD3+ T cells, and F4/80+ macrophages were identified. The presence of tumor infiltrating CD45+ leukocytes increased significantly on day 22 post-tumor implant in mice treated with 4-1BB mAb. There was a time- and dose-dependent increase in intratumoral CD3+ infiltrating T cells after administration of 4-1BB mAb. An increase in tumor infiltrating F4/80+ macrophages was observed on day 22 following 4-1BB mAb administration (Figure 2 and Additional file 1: Table S1).

### 4-1BB agonistic antibody treatment increases circulating cells with a T cell memory phenotype

**Figure 3:**
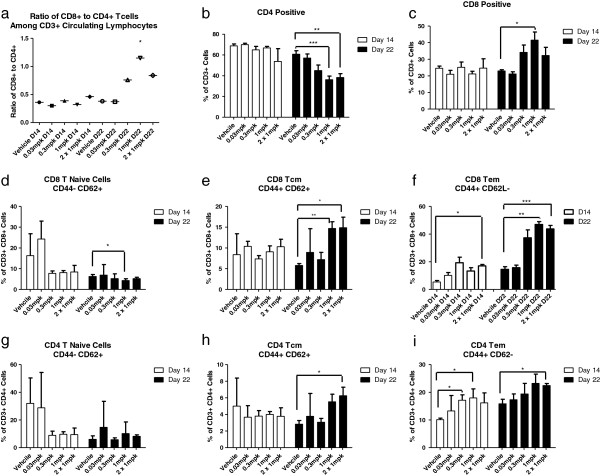
**Effects of 4-1BB agonistic antibody on the T cell functional phenotype.** Peripheral blood mononuclear cells were stained with antibodies to CD3, CD4, CD8, CD44 and CD62L on day 14 or day 22 post-tumor implant. CD3 + CD8+ and CD3 + CD4+ T cells were further evaluated for T central memory (Tcm) (CD44 + CD62L+), T effector memory (Tem) (CD44 + CD62L-), and T naïve (CD44^lo^CD62L^hi^) cell subsets. The ratio of CD8 to CD4 T cells is shown **(a)**. The percentage of CD4 and CD8 T cell populations in treated versus untreated mice is also shown **(a, b, c)** as well as the percentages of the different CD4 and CD8 cell subsets: Tcm **(e, h)**, Tem **(f, i)** and T naïve cells **(d, g)**, represented as a percentage of the parental population gate. *p < 0.05, **p < 0.01, ***p < 0.005 by t-test, n ≥ 3 mice/group, data is representative of 3 independent experiments.

Circulating lymphocytes extracted from peripheral blood of CT26 tumor bearing mice, treated with 4-1BB mAb or vehicle, were characterized for functional T cell subpopulations by surface staining and flow cytometry on days 14 and 22 post-tumor implant (Figure 3). The percentage of T naïve (CD3 + CD44-CD62L+), T central memory (Tcm; CD3 + CD44 + CD62L+) and T effector memory (Tem; CD3 + CD44 + CD62L-.) cell populations was analyzed. No significant change in the ratio of total CD8+ to CD4+ T cells was observed at day 14 post-tumor implant whereas a significant increase was achieved in animals treated with a single dose of 1 mg/kg 4-1BB mAb (p = 0.0193) at day 22 post-tumor implant (Figure 3a). The number of CD4 cells did not change in the treated mice compared to that in the control group. In contrast, a significant increase in the number of CD8+ cells was observed in the mice treated with 0.3 mg/kg and single and double dose of 1 mg/kg 4-1BB mAb (Figure 3b,c). There was a decrease in naïve CD8+ and CD4+ T cells in treated animals versus control. Particularly, a significant decrease in naïve CD8+ T cells was observed in the single 1 mg/kg dose group (p = 0.0126) (Figure 3d,g). In contrast, there was a significant increase in the percentage of circulating CD8+ Tcm cells in mice treated with a single or double dose of 4-1BB mAb versus vehicle from a mean of 5.3% to 14.7% (p = 0.0067) or to 14.8% (p = 0.0334), respectively, on day 22 (Figure 3e). Similar trends were observed in the CD8+ Tem populations. At day 22, increased numbers of Tem cells were observed in both the single and double dose group from a mean of 14.5% to 46.9% (p = 0.0031) or to 43.7% (p = 0.0007), respectively (Figure 3f). A smaller but significant increase in CD4+ Tcm cells was achieved only in double dosed animals from 2.83% to 6.25% (p = 0.0487) on day 22 post-tumor implant (Figure 3h). A significant increase in the percentage of circulating CD4+ Tem cells was also observed at day 14 on the treated mice with 0.3 mg/kg or 1 mg/kg 4-1BB mAb with a change in mean percentage from 10% to 17.1% (p = 0.0374) or 17.9% (p = 0.0165), respectively. At day 22, a significant increase in the CD4+ Tem population was also achieved in the double dose treated group with a mean change from 15.8% to 22.4% (p = 0.0184; Figure 3i).

### [^18^ F] FDG PET visualization of the antitumor responses after 4-1BB treatment

**Figure 4:**
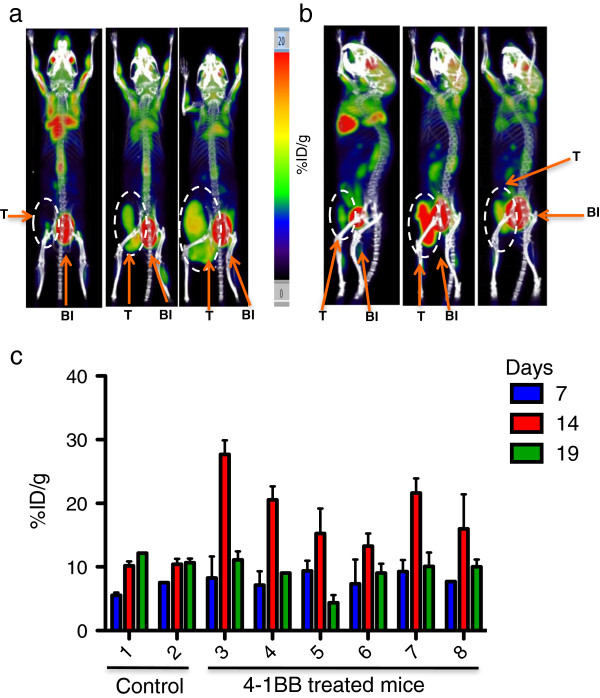
**PET imaging of anti-4-1BB treatment in CT26 tumor-bearing mice.** [^18^ F]FDG microPET imaging showed an increased signal (% ID/g) of [^18^ F]FDG at the tumor site and draining lymph nodes (DLNs) of mice treated with 4-1BB mAb (19.1 ± 2.1 4-1BB mAb group vs. 10.3 ± 0.11 control group, p = 0.06). Representative images shown; orange arrows indicate tumor (T) circled in white, and bladder (Bl). Control mice **(a)** and 4-1BB mAb treated mice **(b)** on day 6 (left), 14 (middle) and 19 (right) post-tumor implant. All mice were treated with 4-1BB mAb or saline on day 9 and 11 post-tumor inoculation. Muscle serves as a background control. **(c)** Standardized uptake values (SUVs) of all the imaged mice. Mouse B is slightly rotated in order for the tumor FDG uptake signal not to overlap with the bladder uptake signal.

Balb/c mice bearing CT26 tumors were imaged by microPET/CT scan before and after two-dose injections of 1 mg/kg 4-1BB mAb or vehicle on days 9 and 11 post-tumor implantation. All mice in this study were imaged on days 7, 14 and 19 post-tumor implant (Figure 4a,b). In replicate experiments, the mice treated with 4-1BB mAb showed an increased signal (% ID/g) of [^18^ F]FDG at the tumor site and draining lymph nodes, while there was no change in the PET tracer accumulation in tumors or draining lymph nodes in control treated mice. The spleen [^18^ F]FDG uptake was also quantified. Five treated mice showed an increase of [^18^ F]FDG uptake signal on day 14 post-treatment whereas no increase in signal was observed in the control mice (data not shown).

### Increased [^18^ F] FDG PET signal after agonistic 4-1BB treatment due to higher tumor leukocyte infiltration as well as higher metabolic activity in infiltrating leukocytes

**Figure 5:**
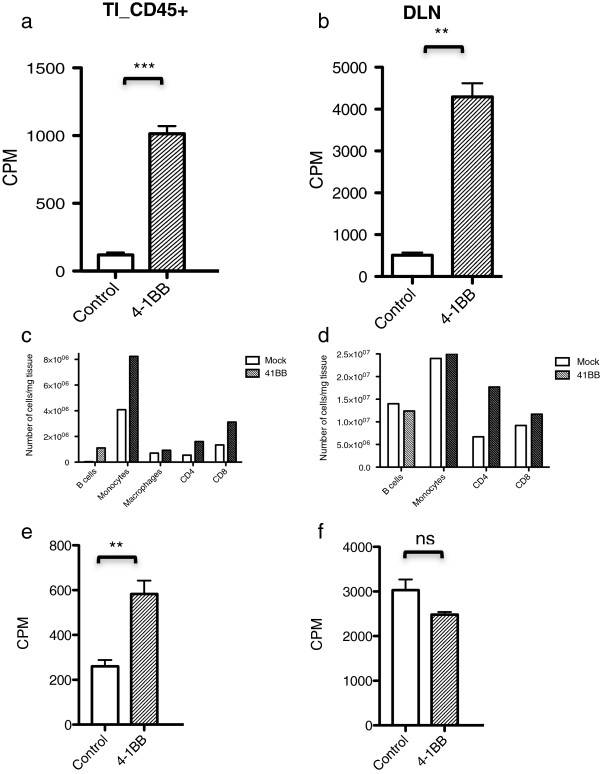
***In vitro *uptake assay of [**^**3**^**H]DDG in cells obtained from mice treated with anti-4-1BB.** Cells were extracted on day 14 post-tumor implant from mice bearing CT26 tumors treated with 4-1BB mAb, which showed an 8-fold increase in non-adherent CD45+ cells extracted from tumors (TI_CD45+; **a)** and draining lymph nodes (DLN; **b)** of mice treated with 4-1BB mAb compared to untreated mice. The TI_CD45+ and DLN cell populations are represented in bar graphs **(c, d)**. **e)***In vitro* co-culture of CT26 cells and splenocytes extracted from the CT26 tumor bearing mice, also showed a significant 2-fold increase in uptake of [^3^H]DDG in splenocytes isolated from the treated mice compared to the untreated mice. CT26 tumor cell uptake did not show a significant difference between the group treated with 4-1BB mAb and the untreated group **(f)**. ** p < 0.01 and *** p < 0.0001 by t-test.

Since the PET [^18^ F]FDG uptake signal from the tumor draining lymph nodes and tumor infiltrating leukocytes overlaps with the tumor signal *in vivo*, we extracted these organs from mice on day 14 post-treatment and performed separate *in vitro* [^3^H]DDG (glucose analogue) uptake assays for each cell type. Tumor CD45+ infiltrating leukocytes (TI_CD45+) and draining lymph nodes (DLNs) were extracted from mice bearing CT26 tumors on day 14 post-tumor implant and the same number of cells was assayed for [^3^H]DDG uptake. There was an 8-fold increase in non-adherent CD45+ cells extracted from tumors and draining lymph nodes of mice treated with 4-1BB mAb compared to untreated mice (Figure 5a,b, p < 0.0001 and p < 0.01, respectively, by t-test). The non-adherent cell populations from tumors and draining lymph nodes were also characterized by flow cytometry (Additional file 2: Figure S1 and Additional file 3: Figure S2). There was a 2-fold increase in monocytes, CD4 and CD8 T cells and a 38-fold increase in B-cells extracted from tumors of mice treated with 4-1BB versus the untreated mice (Figure 5c). A 2.6-fold increase in CD4 T cells extracted from tumor-draining lymph nodes was observed in mice treated with 4-1BB Ab compared to untreated mice (Figure 5d). We also tested these CD45+ cells for *in vitro* [^3^H]DDG uptake after being sorted by flow cytometry. The *in vitro* uptake assay showed a 2-fold increase in uptake of [^3^H]DDG in CD45+ tumor infiltrating cells from mice treated with 4-1BB mAb versus untreated (p < 0.05 by t-test, Additional file 4: Figure S3). Co-culture of CT26 cells and splenocytes extracted from CT26 tumor bearing mice and further *in vitro* uptake assay showed a significant 2-fold increase in uptake of [^3^H]DDG in splenocytes isolated from the mice treated with 4-1BB compared to untreated mice (p < 0.01 by t-test, Figure 5e). Conversely, CT26 tumor cell uptake of [^3^H]DDG did not change between the group treated with 4-1BB mAb and the untreated group (Figure 5f). The tumor cell uptake was 2500-3000 counts per minute (CPM) for both groups. Taken together, we conclude that [^18^ F]FDG PET tracer uptake is due to both an increase in the number and the glycolytic activity of intratumoral lymphocytes upon 4-1BB mAb therapy.

## Discussion

This study used molecular imaging with microPET to characterize the *in vivo* antitumor effects of a 4-1BB agonistic mAb by stimulating an immune response in a mouse model of colon cancer. Similar to results demonstrated with other 4-1BB agonist mAbs [1,7,13], MAB9371 induced CD8 T cell expansion, increased the proportion of CD8 T effector memory populations, and demonstrated tumor regression including long term anti-tumor immune memory. The doses required to achieve anti-tumor responses were significantly lower than those generally reported in scientific literature as a single dose of 0.1 mg/kg was sufficient to cause complete tumor rejection in 60% of animals. Antibody doses of 1 mg/kg or greater resulted in a complete response in nearly all animals. In these studies, [^18^ F] FDG PET imaging allowed efficient monitoring of immune responses induced by 4-1BB. We observed a strong increased mean signal of [^18^ F] FDG at the tumor site and draining lymph nodes in mice treated with 4-1BB mAb compared to untreated mice, which was due to the antitumor immune responses supported by *in vitro* studies. The increased PET signal was due both to a higher metabolic tracer uptake by activated T cells upon 4-1BB stimulation, but also to an increase in the presence of CD45+ T cells within the tumors with 4-1BB treatment, which has been recently characterized as an effect of this mode of therapy [7].

PET imaging is a highly sensitive detection method that has been used widely to image tumors thanks to their increased glycolytic activity, but it could also be an important tool to study immune responses at the whole body level [12]. In particular, it could be of use to study pseudo-progression noted with some forms of immunotherapy (such as the anti-CTLA4 antibody ipilimumab), where some patients may have an initial period of apparent tumor progression before having a response [14]. The [^18^ F] FDG PET tracer is a glucose analog that accumulates inside metabolically active cells, which is routinely used in the clinic as a diagnostic tool for early tumor detection and to assess tumor progression [10]. [^18^ F]FDG has previously allowed detection of activated lymphocytes in the draining lymph nodes and spleen [15] and has provided information on metabolic changes on immune cell activation [16]. Studies in humans have also detected activated lymph nodes during early and asymptomatic HIV infection [17].

Molecular imaging with the cell replication PET tracer 3’-deoxy-3’-^18^ F-fluorothymidine ([^18^ F]FLT) has additionally allowed mapping and noninvasive imaging of cell proliferation in secondary lymphoid organs after CTLA4 blockade with tremelimumab in patients with metastatic melanoma [18], as well as the study of the kinetics of lymphocyte subsets in response to dendritic cell vaccination in melanoma patients with lymph node metastases [19]. Furthermore, the development of new small molecule PET tracers has also allowed visualization of immune cell expansion and activation in the tumor and DLNs in rodents. 1-(2’-deoxy-2’-[^18^F]fluoroarabinofuranosyl) cytosine ([^18^F]FAC) was developed to enable visualization of lymphoid organs and localize immune activation in a mouse model of anti-tumor immunity [20]. Together, these preclinical and early clinical experiences utilizing PET-based molecular imaging support the notion that PET imaging can be used to study T cell responses to cancer. This would allow using a non-invasive imaging approach to determine at which doses there is a pharmacodynamic effect consistent with immune activation. Additionally, differences in visualization of activated immune cells in tumors would allow testing for efficient tumor targeting at those dosing regimens. Therefore, the application of radiolabeled small molecule PET imaging is likely to facilitate the clinical development of immunomodulatory strategies for cancer.

## Conclusions

Anti-41BB therapy induced lymphocyte activation and increased lymphocyte glucose uptake, leading to a higher [^18^ F]FDG accumulation at the tumor and draining lymph nodes detectable by microPET scanning. This non-invasive imaging approach makes the visualization of these 4-1BB agonistic antibody-induced antitumor immune responses possible, providing an efficient means to guide the clinical development of these antibodies in patients.

## Methods

### Animals and cell lines

Balb/c mice were purchased from Charles River Laboratories International, Inc. The procedures were carried out in accordance with the University of California Los Angeles (UCLA) animal care policy, with the Animal Research Committee approval (ARC protocol # 2004-159-21), and the Institutional Animal Care and Use Committee (IACUC) guidelines at Pfizer (La Jolla, CA). All handling and procedures were documented on an IACUC approved Animal Use Protocol (AUP). The tumor cell lines of Balb/c origin, CT26, a murine colon carcinoma, and 4 T1, a highly metastatic murine mammary carcinoma, were obtained from American Type Culture Collection (ATCC, Manassas, VA) and cultured according to the protocols provided by ATCC.

### Subcutaneous tumor models in mice

One million CT26 or fifty thousand 4 T1 cells were injected subcutaneously into wild type Balb/c mice. Six to seven days post-tumor implant, mice were randomized according to tumor volume, which on average was 150 mm^3^. After randomization, saline or 4-1BB antibody (catalog #MAB9371, clone #158321, R&D Systems; Minneapolis, MN) was dosed ranging from 1 mg/kg to 0.001 mg/kg diluted in PBS, and administered intraperitoneally (i.p), as a single dose on day 9, or double dose on days 9 and 11 post-tumor implant. Mice that had been tumor-free for over 45 days were re-challenged by implanting CT26 on the right flank and 4 T1 tumors on the left flank. Tumor volume (mm^3^) and body weight were recorded two or three times per week using Preisser digital calipers (Flexbar Machine Company, Islandia, NY). Tumor volume was calculated as [length × (width x width)] × 0.5 = volume in mm^3^. Animals were euthanized according to IACUC guidelines when the tumor volume reached 2,000 mm^3^. Depending upon the individual study goals, peripheral blood or serum was collected upon euthanasia, as outlined by the Pfizer La Jolla, IACUC guidelines. Tumor sections were preserved in OCT or 10% paraformaldehyde for histological and immunohistochemical (IHC) analysis.

### Immune cell subset depletion experiments

Balb/c mice with 7-day established CT26 tumors were randomized to receive i.p. injection with 100 μg of anti-mouse CD8 (clone YTS169.4, Bio-X-Cell, West Lebanon, NH), 100 μg of anti-mouse CD4 (clone GK1.5, Bio-X-Cell) or 100 μg anti-asialoGM1 (Wako Chemicals; Richmond, VA). Antibody depletion injections were repeated weekly. For macrophage depletion, 2 mg of clodronate liposomes (purchased from Dr. N. van Rooijen, Clodronate Liposomes Foundation, Haarlen, Netherlands) were injected i.p. on day 7, and subsequent injections were repeated at weekly intervals using 1 mg per injection.

### Micro-PET/computed tomography imaging

Mice were anesthetized with 2% isoflurane and injected intravenously with 200 μCi of [^18^ F]FDG. Following one-hour uptake, mice were placed in an imaging chamber for sequential imaging with a microPET Focus 220 and microCAT II CT scanner (Siemens Preclinical Solutions, Washington D.C.). MicroPET data were acquired for 10 minutes and reconstructed and analyzed after using OsiriX Imaging Software (Pixmeo, Geneva, Switzerland). The mean intensity of the region of interest (ROI), based on the percent-injected dose per gram, was normalized to a background ROI drawn around muscle in the same animal.

### Flow cytometry analysis of immune cells

Animals were euthanized and blood was collected via intra-cardiac puncture following IACUC guidelines. Lymphocytes were isolated from whole blood using Lympholyte Mammal (Cedarlane Laboratories, Burlington, NC) following the manufacturer’s instructions. Approximately 3-5 × 10^5^ cells per sample were stained with CD3ϵ_APC, CD4_PE, or CD8α_PerCP_Cy5.5 antibodies (Biolegend, San Diego, CA). Tumors and draining lymph nodes were also extracted 14 days post-tumor implant, subsequently minced with surgical scissors and placed on a rotator in collagenase-D (Roche, San Francisco, CA) and DNAse-1 type IV (Sigma, St. Louis, MO) for one hour at 37^o^C. Single-cell suspensions were stained with fluorochrome-conjugated antibodies to CD62L_Alexa700, CD44_APC_Cy7, CD4_APC_Cy7, CD8_APC_Cy7, CD3_eFluor450, CD14_APC, CD11b_PE, CD19_eFluor_450, CD28_APC, CD45_FITC and CD32/16 (eBiosciences, San Diego, CA), or CD27_PE_Cy7 and F4/80_PE (Biolegend). All data were collected on a LSRII (Becton Dickinson, Franklin Lakes, NJ) or FACSCalibur (Becton Dickinson) and analysis was performed using FlowJo software (TreeStar Inc., San Carlos, CA).

### [^3^H]DDG uptake *in vitro*

The same single cell suspensions used for flow cytometric analysis and extracted from tumors and draining lymph nodes on day 14 post-tumor implant, were also prepared for an uptake assay with [^3^H]DDG, an analog of [^18^ F]FDG, obtained from Movareck Biochemicals and Radiochemicals (Brea, CA). Cells were counted and placed on 100 mm petri dishes for 3-4 hours, allowing cell attachment of tumor cells. Cells from the spleen of the same mice were also extracted on day 14 and co-cultured with CT26 cells for 24 hours. Non-adherent CD45+ infiltrating cells from the tumor, draining lymph nodes and spleen, were counted and seeded on 24 well plates, 1 × 10^5^ cells/well, in glucose-free media (5% FBS). The assay was performed in triplicates. 100 μCi [^3^H]DDG was added to the cells and the mixture incubated at 37^o^C for 2 hours. Cells were subsequently washed with glucose-free and serum-free media four times and then placed in wells of a 96-well 0.22-μm multiscreen filter bottom plate (Millipore, Hayward, CA). Washed plates were dried and 100 μl of scintillation fluid was added to each well. Accumulated radioactivity was counted using a BetaMax plate reader (PerkinElmer, Waltham, MA). The same cells extracted on day 14 post-tumor implant were also stained with FITC_CD45+ Ab and sorted automatically with a FACS ARIA II Cell Sorter (Becton Dickinson).

### Immunohistochemistry, immunofluorescence and image analysis

Immunohistochemical staining was performed on frozen, OCT-embedded, tumor samples using the automated Leica Bonds Max system (Leica, Buffalo Grove, IL). Five micron, cryostat sections were air-dried, fixed in chilled acetone and 95% ethanol, washed in Bonds wash solution (Leica) blocked (Dako block, Dako, Glostrup Denmark) and stained with biotinylated anti-mouse CD3 antibody (R&D systems) for 20 minutes at a 1:2000 dilution. The sections were then incubated with Streptavidin-HRP (Leica) and immune complexes were visualized using DAB substrate with hematoxylin counterstain.

Immunofluoresence staining was performed on frozen, OCT-embedded, tumor samples using the automated Leica Bonds Max system. Five micron cryostat sections were air-dried, then fixed in a 1:1 ratio of chilled acetone and 95% ethanol for 5 minutes. Following fixation, sections were washed in Bonds wash solution, blocked and stained with primary antibodies to CD45 (BD Pharmingen) and F4/80 (Thermo Scientific) for 60 minutes at a 1:1500 dilution. The sections were then incubated with the secondary antibody Alexa Fluor 594 1:200 (Invitrogen) and coverslipped with fluoresecent mounting media with DAPI counterstain.

For image analysis, up to 40 representative images at 20x magnification were multispectrally captured on the Vectra™ system (Malleswaram, Banglore). Using Nuance™ (Sidney, Australia) appropriate spectral libraries with the correct background threshold were created from each image. Samples were evaluated using Inform™ software (Chicago, IL). Regions of interest were defined by a single algorithm that classified functional regions such as, viable tumor, non-tumor and background. The algorithm for CD3 also included a cell count segmentation component, which determined the CD3 positive cell count. Two data set calculations were formulated for CD3. One represents the number of CD3 positive cell count per 100,000 pixels of viable tumor area and the other represents the pixel area of CD3 positive cells per 100 pixels of viable tumor area. The pixel area of CD3 positive cells was also calculated in order to accurately compare CD3 data to the CD45 and F4/80 data, which was calculated based on positive pixel area and not cell count. CD45 positivity was evaluated based on CD45 pixel area per viable pixel area. Data represents the pixel area of CD45 positive cell area per 100 pixels of viable tumor area. F4/80 positivity was evaluated based on F4/80 pixel area per viable pixel area. Data represents the pixel area of F4/80 positive cell area per 100 pixels of viable tumor area.

### Statistical analysis

Data were analyzed with GraphPad Software (La Jolla, CA). Significance was determined using ANOVA or student t-test with two-tailed p-values. Excel and Prism GraphPad were used to evaluate the numerical data generated by Inform for the IHC image analyses. P-values were calculated based on a one-tailed t-test and means of significance determined by P < 0.05.

## Abbreviations

UCLA: University of California Los Angeles; JCCC: Jonsson Comprehensive Cancer Center; PET: Positron emission tomography; mAb: Monoclonal antibodies; [18 F]FDG: [^18^ F]-Labeled fluoro-2-deoxy-2-D-glucose; TNFRSF: Tumor necrosis factor receptor superfamily; DC: Dendritic cells; NK: natural killer cells; NKT: Natural killer T cells; APCs: Antigen-presenting cells; TGI: Tumor growth inhibition; Tcm: T central memory; Tem: T effector memory; TI_CD45+: Tumor infiltrating leukocytes; DLNs: Draining lymph nodes; [18 F]FLT: 3’-Deoxy-3’-^18^ F-fluorothymidine; [18 F]FAC: 1-(2’-Deoxy-2’-[18 F]fluoroarabinofuranosyl) cytosine; i.p: Intraperitoneally; IHC: Immunohistochemical; ROI: Region of interest.

## Competing interest

Mark W. Elliott, Michelle Lee and Leslie L. Sharp were employees of Pfizer Inc. at the time of this work, a company that has an interest in the clinical development of anti-CD137 activating antibodies.

## Authors’ contributions

HE-O and MWE performed most of the studies, with the significant contribution by MA, ML and CN. HE-O, BC-A, EM-R, EA performed flow cytometry analyses. HE-O, LW and CR performed the PET imaging studies and their interpretation. HE-O, MWE, LLS and AR designed the studies and wrote the manuscript. All authors read and approved the final version.

## Supplementary Material

Additional file 1: Table S1 Treatment with 4-1BB agonistic antibody increases tumor infiltrating leukocytes. This table provides further details regarding the immunohistochemical and immunofluorescence data shown in Figure 2. Samples are divided in groups on the basis of their cell type (CD3+, CD45+ and F4/80+) and 4-1BB dose treatment (1 mg/kg ×2 = double dose of 4-1BB mAb on day 9 and 11; ×1 = single dose). The cell expression was analyzed on day 14 and day 22 post-tumor implant and is scored as NS (not significant), medium (++) or high (+++) based on the pixel area of positively stained cells per 100 pixel area of viable tumor. The P values and number of mice (n) per group are also shown. Click here for file

Additional file 2: Figure S1 Flow cytometric analysis of CD45+ cells extracted from tumors at day 14 post-tumor implant. Tumor cells extracted from untreated mice (a) and mice treated with 1 mg/kg of 4-1BB mAb (b) were stained with the fluorochrome-conjugated antibodies to CD62L_Alexa700, CD44_APC_Cy7, CD4_APC_Cy7, CD8_APC_Cy7, CD3_eFluor450, CD14_APC, CD11b_PE, CD19_eFluor_450, CD28_APC, CD45_FITC, CD27_PE_Cy7 and F4/80_PE. Cell types gated are specified on each graph and percentages shown. Click here for file

Additional file 3: Figure S2 Flow cytometric analysis of CD45+ cells extracted from draining lymph nodes at day 14 post-tumor implant. Lymph node cells extracted from untreated mice (a) and mice treated with 1 mg/kg of 4-1BB mAb (b) were stained with the fluorochrome-conjugated antibodies to CD62L_Alexa700, CD44_APC_Cy7, CD4_APC_Cy7, CD8_APC_Cy7, CD3_eFluor450, CD14_APC, CD11b_PE, CD19_eFluor_450, CD28_APC, CD45_FITC, CD27_PE_Cy7 and F4/80_PE. Cell types gated are specified on each graph and percentages shown. Click here for file

Additional file 4: Figure S3 *In vitro* uptake assay of CD45+ cells sorted from CT26 tumors extracted on day 14 post-tumor implant, showed a 2-fold increase in uptake of [^3^H]DDG in CD45+ tumor infiltrating cells from mice treated with 4-1BB mAb (*p < 0.05 by Student’s t-test). Click here for file
