# Supplementary material for: PET imaging to non-invasively study immune activation leading to antitumor responses with a 4-1BB agonistic antibody
Source: J Immunother Cancer. 2013 Aug 27;1:14. doi: 10.1186/2051-1426-1-14 (PMC4019904; doi:10.1186/2051-1426-1-14)
Supplement: Additional file 1: Table S1 — Treatment with 4-1BB agonistic antibody increases tumor infiltrating leukocytes. This table provides further details regarding the immunohistochemical and immunofluorescence data shown in Figure 2. Samples are divided in groups on the basis of their cell type (CD3+, CD45+ and F4/80+) and 4-1BB dose treatment (1 mg/kg ×2 = double dose of 4-1BB mAb on day 9 and 11; ×1 = single dose). The cell expression was analyzed on day 14 and day 22 post-tumor implant and is scored as NS (not significant), medium (++) or high (+++) based on the pixel area of positively stained cells per 100 pixel area of viable tumor. The P values and number of mice (n) per group are also shown. [file 2051-1426-1-14-S1.doc]

**Additional file 1: Table S1.** Treatment with 4-1BB agonistic antibody increases tumor infiltrating leukocytes. This table provides further details regarding the immunohistochemical and immunoflurecence data shown in Figure 2. Samples are divided in groups on the basis of their cell type (CD3+, CD45+ and F4/80+) and 4-1BB dose treatment (1 mg/kg x2 = double dose of 4-1BB mAb on day 9 and 11; x1= single dose). The cell expression was analyzed on day 14 and day 22 post-tumor implant and is scored as NS (not significant), medium (++) or high (+++) based on the pixel area of positively stained cells per 100 pixel area of viable tumor. The P values and number of mice (n) per group are also shown.

|  | Study Day |  | 14 |  |  | 22 |  |
| --- | --- | --- | --- | --- | --- | --- | --- |
|  | 4-1BB mAb dose | 0.3 mg/kg  x 1 | 1 mg/kg  x 1 | 1 mg/kg  x 2 | 0.3 mg/kg  x 1 | 1 mg/kg  x 1 | 1 mg/kg  x 2 |
| CD3+ |  | NS | NS | ++ p<0.006  N=6 | ++ p<0.01  N=3 | ++ p<0.0001  N=3 | +++ p<0.001  N=5 |
| CD45+ |  | NS | NS | NS | +++ p<0.002  N=5 | +++ p<0.001  N=3 | +++ p<0.0009  N=4 |
| F4/80+ |  | NS | NS | NS | NS | +++ p<0.0002  N=3 | +++ p<0.0002  N=3 |

Legend: NS: not significant
