# Supplementary material for: PET imaging to non-invasively study immune activation leading to antitumor responses with a 4-1BB agonistic antibody
Source: J Immunother Cancer. 2013 Aug 27;1:14. doi: 10.1186/2051-1426-1-14 (PMC4019904; doi:10.1186/2051-1426-1-14)
Supplement: Additional file 2: Figure S1 — Flow cytometric analysis of CD45+ cells extracted from tumors at day 14 post-tumor implant. Tumor cells extracted from untreated mice (a) and mice treated with 1 mg/kg of 4-1BB mAb (b) were stained with the fluorochrome-conjugated antibodies to CD62L_Alexa700, CD44_APC_Cy7, CD4_APC_Cy7, CD8_APC_Cy7, CD3_eFluor450, CD14_APC, CD11b_PE, CD19_eFluor_450, CD28_APC, CD45_FITC, CD27_PE_Cy7 and F4/80_PE. Cell types gated are specified on each graph and percentages shown. [file 2051-1426-1-14-S2.pptx]

## Slide 1
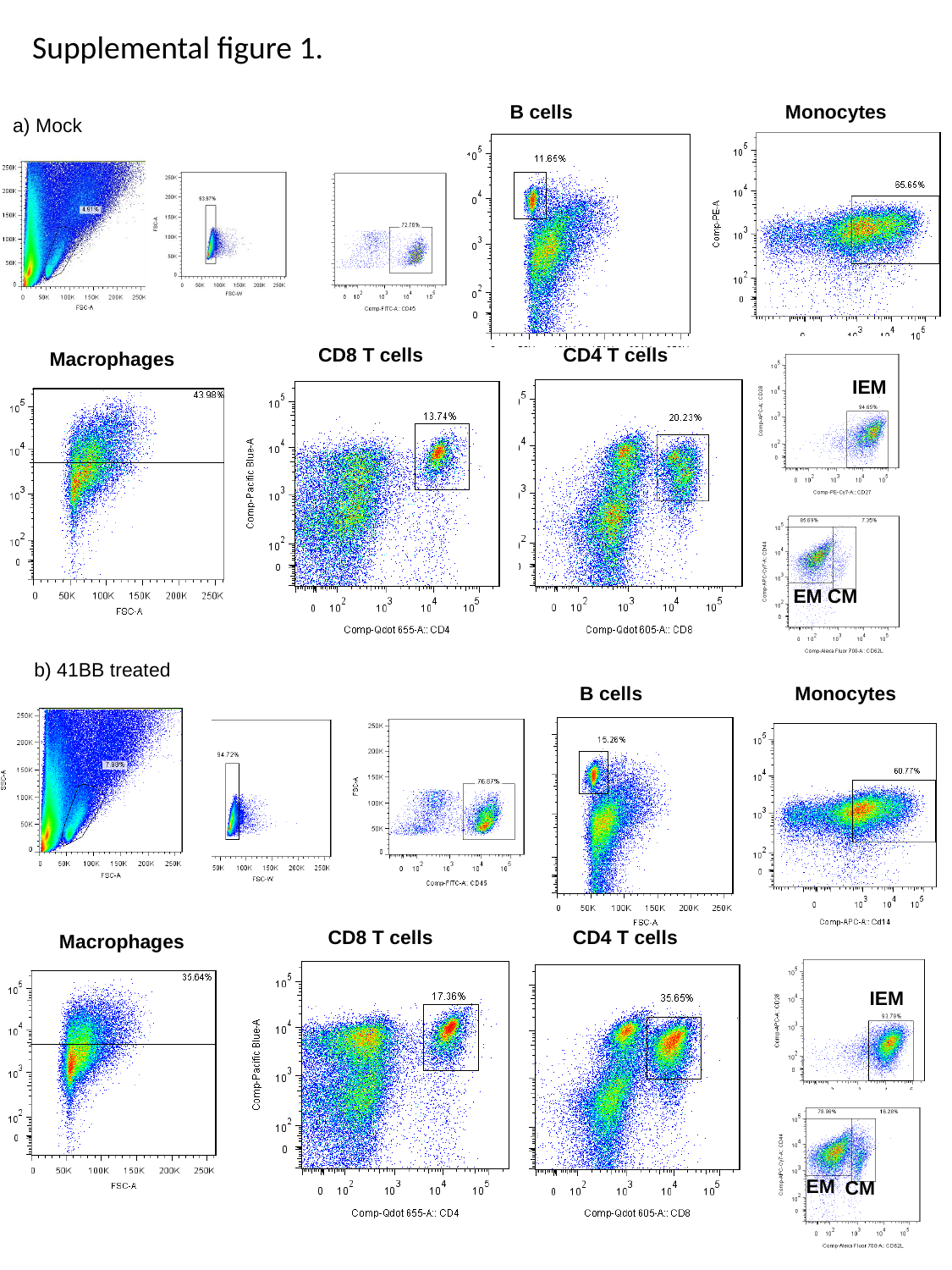

Supplemental figure 1.
B cells
Monocytes
a) Mock
CD8 T cells
CD4 T cells
Macrophages
IEM
EM
CM
b) 41BB treated
B cells
Monocytes
CD8 T cells
CD4 T cells
Macrophages
IEM
EM
CM
