# Supplementary material for: PET imaging to non-invasively study immune activation leading to antitumor responses with a 4-1BB agonistic antibody
Source: J Immunother Cancer. 2013 Aug 27;1:14. doi: 10.1186/2051-1426-1-14 (PMC4019904; doi:10.1186/2051-1426-1-14)
Supplement: Additional file 3: Figure S2 — Flow cytometric analysis of CD45+ cells extracted from draining lymph nodes at day 14 post-tumor implant. Lymph node cells extracted from untreated mice (a) and mice treated with 1 mg/kg of 4-1BB mAb (b) were stained with the fluorochrome-conjugated antibodies to CD62L_Alexa700, CD44_APC_Cy7, CD4_APC_Cy7, CD8_APC_Cy7, CD3_eFluor450, CD14_APC, CD11b_PE, CD19_eFluor_450, CD28_APC, CD45_FITC, CD27_PE_Cy7 and F4/80_PE. Cell types gated are specified on each graph and percentages shown. [file 2051-1426-1-14-S3.pptx]

## Slide 1
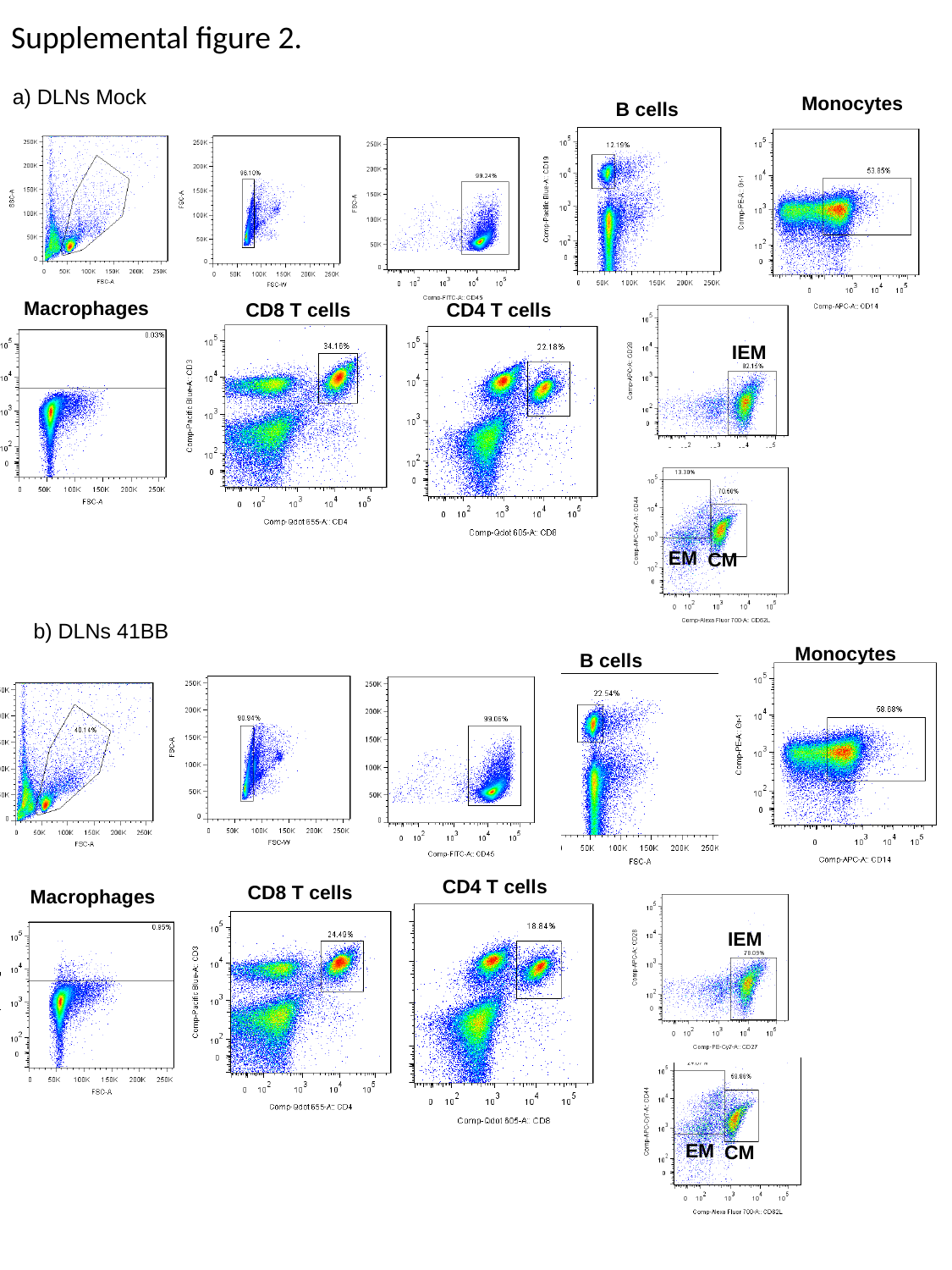

Supplemental figure 2.
a) DLNs Mock
Monocytes
B cells
Macrophages
CD8 T cells
CD4 T cells
IEM
EM
CM
b) DLNs 41BB
Monocytes
B cells
CD4 T cells
CD8 T cells
Macrophages
IEM
EM
CM
