# Supplementary figures and images for: PET imaging to non-invasively study immune activation leading to antitumor responses with a 4-1BB agonistic antibody
Source: J Immunother Cancer. 2013 Aug 27;1:14. doi: 10.1186/2051-1426-1-14 (PMC4019904; doi:10.1186/2051-1426-1-14)

## Slide 1
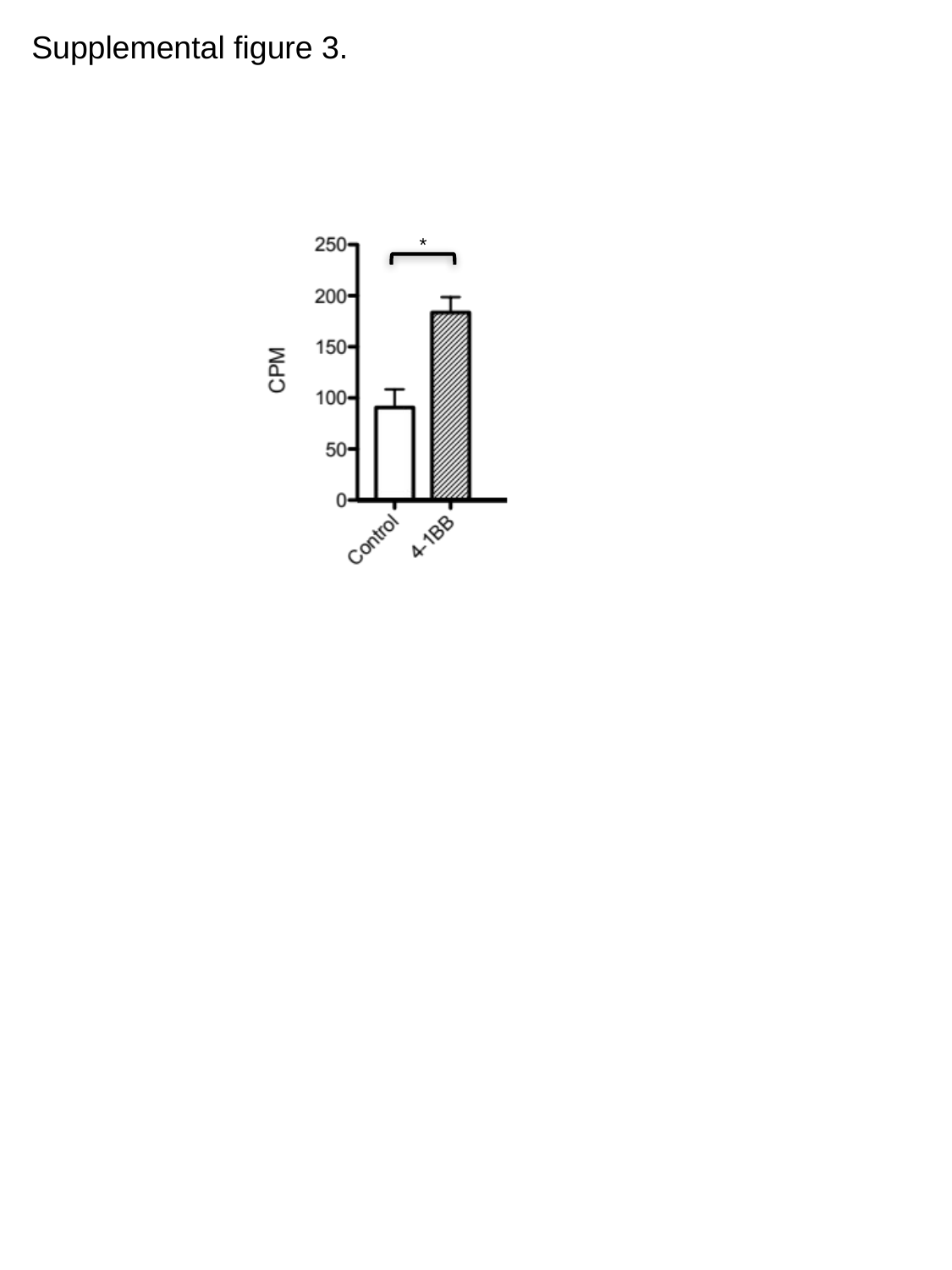

Supplemental figure 3.
*

Supplement: Additional file 4: Figure S3 — In vitro uptake assay of CD45+ cells sorted from CT26 tumors extracted on day 14 post-tumor implant, showed a 2-fold increase in uptake of [3H]DDG in CD45+ tumor infiltrating cells from mice treated with 4-1BB mAb (*p < 0.05 by Student’s t-test). [file 2051-1426-1-14-S4.pptx]
